# Effect of a Modified Preparation Design on the Marginal and Internal Fit of 3D-Printed Onlays: A Feasibility Study

**DOI:** 10.1155/ijod/7599339

**Published:** 2025-09-15

**Authors:** Stephanie Ung, Arthi Veerasamy, Wendy-Ann Jansen van Vuuren, Vincent Bennani

**Affiliations:** ^1^Sir John Walsh Research Institute, University of Otago, Dunedin, New Zealand; ^2^Department of Oral Rehabilitation, Faculty of Dentistry, University of Otago, Dunedin, New Zealand

**Keywords:** cement gap, computer-aided design, marginal adaptation, onlay preparation, printing, tooth preparation

## Abstract

**Objective:** Bonded onlay preparations provide minimal guidance during cementation, which may compromise seating accuracy and increase marginal and internal discrepancies, potentially affecting restoration longevity. This study aims to assess whether a modified preparation (MP) design improves onlay positioning during cementation to enhance marginal and internal fit.

**Materials and Methods:** The conventional preparation (CP) design was prepared on a typodont tooth, scanned, and replicated (*n* = 20). A cross-shaped groove was added to the prepared typodont tooth to create the MP design, then scanned and replicated (*n* = 20). For both groups, a restoration was designed using computer-aided design (CAD) and computer-aided manufacturing (CAM). Each onlay was three-dimensionally (3D) printed, assessed for fit, and then bonded onto the replicas. Ten specimens from each group were sectioned horizontally and 10 vertically. Absolute marginal discrepancy (AMD) and internal cement thickness (ICT) were measured on the horizontally and vertically sectioned specimens under a stereomicroscope. Data were analyzed using the Mann–Whitney *U* test (*α* = 0.05).

**Results:** The MP showed a statistically significant lower mean AMD than the CP at the palatal (*p*=0.035) and buccal (*p*=0.006) sites. However, there was no statistically significant difference at the mesial site (*p*=0.193). The CP had a statistically significant lower ICT than the MP in the vertical section (*p*=0.009), but the difference in the horizontal section was not statistically significant (*p*=0.121).

**Conclusions:** The MP improved AMD compared to the CP at buccal and palatal sites, but increased ICT in the vertical section.

**Clinical Significance:** The MP design showed that a minor modification to the conventional bonded onlay preparation design improved AMD but increased ICT. These findings guide clinicians in selecting optimal designs to improve restoration adaptation and longevity.

## 1. Introduction

**Figure 1:**
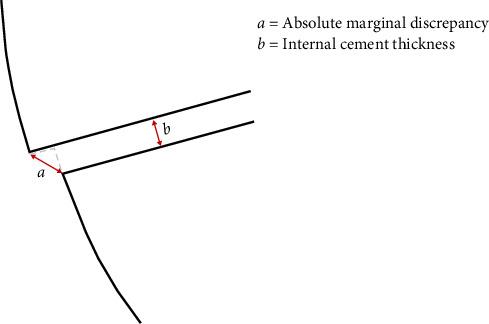
Schematic showing how AMD and ICT were measured.

Indirect partial coverage restorations are conservative and esthetic treatment options for restoring teeth with significant loss of tooth structure. The marginal and internal fit is critical for the clinical longevity of indirect restorations, as poor adaptation can result in secondary caries, debonding, and mechanical failure [1, 2]. Marginal fit can be quantitatively measured as absolute marginal discrepancy (AMD), which is defined as the distance between the cavosurface angle of the preparation to the margin of the restoration [3], as shown in Figure 1. Increased AMD facilitates plaque accumulation and microleakage, resulting in secondary caries, endodontic complications, and periodontal disease. Additionally, it can cause cement degradation, potentially leading to debonding of the restoration or discoloration of the margin, causing both functional and esthetic concerns [1, 2, 4–6]. Internal gap is defined as the perpendicular distance from the internal surface of the restoration to the axial wall of the preparation [3], as shown in Figure 1, and can be quantitatively measured as internal cement thickness (ICT). Excessive ICT can reduce fracture resistance and retentive bond strength, increasing the risk of restoration failure [6–8]. Therefore, achieving optimal marginal and internal fit is essential for the long-term success of restorations.

Preparation design has been shown to influence the fit of indirect restorations [9–13]. Compared to full-coverage crowns, bonded onlays are more susceptible to misfit during cementation due to their complex preparation design. This often includes increased taper, flatter cuspal morphology, shallow occlusal and proximal boxes, and the absence of retentive features. These features provide limited lateral stability; therefore, the restoration lacks a defined path of insertion, increasing the risk of seating errors during cementation [14, 15]. Their complex geometry may also affect the accuracy of intraoral scans [16], resulting in poor marginal and internal fit.

Marginal and internal fit can be evaluated using various methods, including the direct view method, replica technique, microcomputed tomography (µCT), and cross-sectioning [17, 18]. While the direct view and replica techniques are nondestructive, they have limitations in accuracy and precision when assessing internal gaps [15]. The use of µCT allows for three-dimensional (3D) analysis but is costly and may lack the resolution for fine details [19]. The present study measured AMD and ICT using two-dimensional (2D) sectioning and direct viewing under an optical stereomicroscope. Cross-sectioning remains widely used to provide precise, direct measurements of AMD and ICT. This method produces results that accurately reflect the observed increases in marginal gap size following cementation [9, 12, 20].

Computer-aided design (CAD) and computer-aided manufacturing (CAM) systems are becoming increasingly popular for the fabrication of indirect restorations. While widely used, subtractive manufacturing has limitations in replicating fine surface details, sharp internal angles, and deep groove regions due to constraints in the size of the milling burs, often resulting in enlarged internal gaps [10, 11, 21]. Additive manufacturing (3D printing) involves creating objects from digital designs by depositing material in incremental layers until the object is completed [21]. In contrast to subtractive manufacturing, 3D printing can produce highly complex shapes, as it is not limited by the size and shape of the milling burs [21, 22]. However, the marginal and internal fit of 3D-printed restorations may be influenced by the printing technology, device, printing parameters, and processing methods [22–25].

Despite the growing use of 3D-printed restorations in clinical practice, limited research has explored the effect of preparation design on marginal and internal fit [26]. Existing studies primarily focus on milled restorations or do not adequately address the influence of preparation geometry on the fit of 3D-printed onlays. This study aimed to evaluate the effect of two onlay preparation designs on AMD and ICT. The modified preparation (MP) design introduced a cross-shaped occlusal groove into the conventional preparation (CP) design. This feature was hypothesized to provide resistance to movement in three planes, including mesio-distal, bucco-palatal, and rotational displacement. The dimensions were selected based on clinical principles of conservative occlusal reduction [27] and the limitations in the size of commonly used diamond burs for tooth preparation. The null hypothesis was that preparation design would not affect the marginal and internal fit of the 3D-printed onlays.

## 2. Materials and Methods

**Figure 2:**
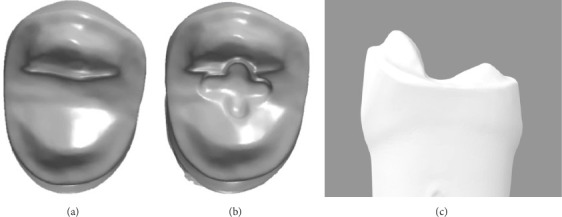
Occlusal view of preparation designs on 3Shape Dental Manager 2023 software. (A) Conventional preparation. (B) Modified preparation. (C) View of preparation from distal surface.

**Figure 3:**
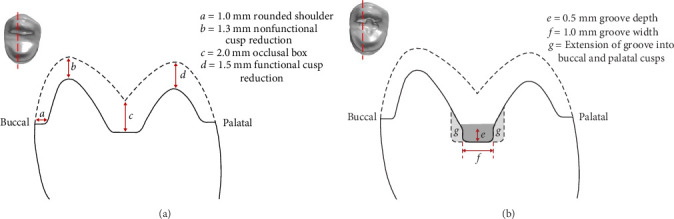
Schematic of preparation designs. (A) Conventional preparation. (B) Modified preparation.

The CP was prepared on a right maxillary first premolar typodont tooth (A5AN-500-#14; Nissin Dental Products, Kyoto, Japan) under water irrigation, using a dental handpiece (T2 Racer Air-Driven Handpiece; Dentsply Sirona, Charlotte, NC, USA) and diamond burs (NTI Diamond Bur Friction Grip Fine 856-018F; Kerr, Brea, CA, USA). The CP followed a tabletop onlay design, characterized by a flat occlusal surface with minimal axial reduction. The preparation had a 1.0 mm rounded shoulder, a 2.0 mm occlusal box, 1.5 mm reduction on the functional cusp (palatal), and 1.3 mm reduction on the nonfunctional cusp (buccal). As shown in Figures 2A, C and 3A, the shallow occlusal box and cusps provide minimal guidance during seating.

The sample size was determined from a previous study investigating the effect of preparation design on the marginal and internal fit of veneers fabricated using CAD/CAM [28]. The study reported large effect sizes ranging from 0.266 to 0.600 across marginal and internal gap parameters. Using these findings, the sample size of 20 specimens per group was selected to ensure sufficient statistical power for determining clinically significant differences in AMD and ICT between the CP and MP designs. The sample size was chosen to ensure at least 80% power to detect a clinically significant difference in AMD and ICT at a 5% significance level (*α* = 0.05), assuming a medium effect size (Cohen's *d* ≈ 0.5).

To design the onlay restorations for the CP, the preparation was cleaned using an air–water syringe and scanned using an intraoral scanner (CEREC Primescan, v5.2, Dentsply Sirona, Charlotte, NC, USA).

To replicate the CP preparation, a silicone impression material (Wirosil; BEGO, Bremen, Germany) was used and then poured with an inert and dimensionally stable resin die material (Exakto-Form; Bredent, Senden, Germany), which is reported to provide a homogeneous and uniform bonding surface [29–31].

To maintain consistency in the preparation designs, the same typodont tooth prepared with the CP was used to create the MP. A cross-shaped groove of depth 0.5 mm and width 1.0 mm was added in the center of the occlusal surface to act as a positioning guide, as shown in Figures 2B and 3B. The rest of the preparation remained unchanged. The MP was scanned and replicated as previously described.

The intraoral scan data were exported to dental software (3Shape Dental Manager 2023, v2.102.1.1; 3Shape, Copenhagen, Denmark) as standard tessellation (STL) files in the highest resolution. An onlay restoration was designed for the CP and then copied to the MP, ensuring both abutments received the same design. The cement spacer was set to 0 µm [32], with an overall restoration minimal thickness set at 0.5 mm. The designs were exported as STL files to a 3D printing software (Asiga Composer 1.2; Asiga, Sydney, Australia) and 3D-printed using a methacrylate-based material (Freeprint temp 385; Detax). The postprinting processing method was selected according to previous studies, which produced restorations with the best fit [23] and accuracy [33]. Following printing, the printer build-plate was removed, and the onlays were submerged in a container filled with clean 99.8% isopropyl alcohol. The container was placed in an ultrasonic bath (Ultrasonic Cleaner; Daihan Scientific, Seoul, South Korea) for 10 min, then repeated in new and clean 99.8% isopropyl alcohol for 10 min. The onlays were carefully scraped from the build-plate, dried with clean and compressed air, and light-cured for 2000 flashes under a closed-chamber curing light (OtoFlash G171; VOCO, Cuxhaven, Germany). The supports were removed using a diamond bur (Komet Diamond Bur 8379-023; Komet, Fort Mill, SC, USA) and a high-speed handpiece (T2 Racer Air-Driven Handpiece; Dentsply Sirona, Charlotte, NC, USA).

**Figure 4:**
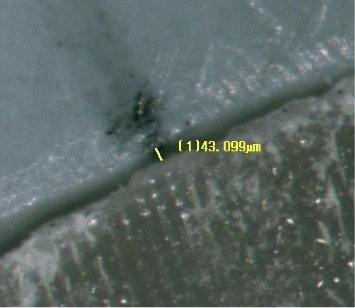
Initial microscopic evaluation of marginal gap.

**Figure 5:**
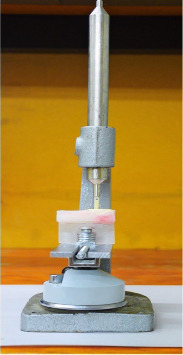
Custom-made seating device used for cementation of all onlays.

To ensure the dimensional accuracy of the onlays, multiple measurements were taken with a caliper (Iwanson for Metal; ASA Dental, Henry Schein, Australia) and compared for consistency. The onlays were visually inspected on the replicas to ensure a good fit and stable model adaptation. Before cementation, a preliminary assessment of the marginal gap was performed under finger pressure and a stereomicroscope (Nikon SMZ800N; Nikon, Tokyo, Japan) to ensure no marginal gap exceeded 50 µm. An example of this measurement is shown in Figure 4. None of the restorations required adjustments during the preliminary assessment. This assessment confirmed that both groups had consistent precementation adaptation. In preparation for cementation, the onlays were cleaned using 50% isopropyl alcohol and dried with gentle and oil-free air flow for 5 s. An adhesive (Scotchbond Universal Plus Adhesive; 3M, St. Paul, MN, USA) was applied to the intaglio surface. Excess adhesive was removed with gentle and oil-free airflow for 5 s. The onlays were light-cured with a polymerization light (Bluephase; Ivoclar Vivadent, Schaan, Liechtenstein) on 650 mW/cm^2^ mode for 10 s. A self-adhesive resin cement (RelyX Unicem 2 Automix; 3M, St. Paul, MN, USA) was applied onto the intaglio surfaces of the onlays, with the amount of cement standardized by dispensing 2 mm of the syringe for each onlay. The onlays were positioned onto the abutment teeth and cemented using a custom-made device. The device (Figure 5) consisted of a sliding weighted vertical arm with a silicone tip that applied uniform pressure onto the occlusal surface of the onlay during the bonding procedure, standardizing the seating force and direction across all specimens and minimizing operator-induced variability. Excess cement was removed using an applicator brush (Microbrush; Microbrush International), followed by light-curing each surface with a polymerization light on 650 mW/cm^2^ mode (Bluephase; Ivoclar AG) for 20 s. The specimens were left undisturbed for an additional 6 min under the custom-made device to allow complete autopolymerization of the cement. Following cementation, the specimens were placed in distilled water at room temperature, which was controlled at 23°C for 24 h. For standardization, the same operator (Stephanie Ung) bonded all 40 onlays.

**Figure 6:**
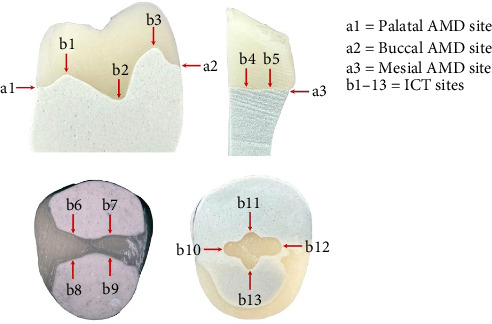
Measurement sites for AMD and ICT.

Half of the CP specimens (*n* = 10) were sectioned in the vertical (vestibulo–oral) plane, while the remaining were sectioned in the horizontal (transverse) plane. For the vertical sections, the distal half of the specimen was used for the measurement of AMD at buccal and palatal sites and three ICT sites (Figure 6). The mesial segment was sectioned in half again to allow AMD measurement at the mesial site, and two ICT sites (Figure 6). The same was completed for MP specimens. To ensure consistent sectioning in horizontal and vertical planes, two silicone keys were fabricated using silicone putty (Sheratandem 85; SHERA Werkstoff-Technologie, Lemförde, Germany) to mark the specimens before sectioning.

The onlay specimens were sectioned using a slow-speed precision diamond saw (LECO VC-50 Model 801-900; LECO, St. Joseph, MI, USA) with a 0.38 mm-thick diamond blade under constant water irrigation. After sectioning, the specimens were polished using 0.05 µm grain size discs (LaboPol-21; Struers, Ballerup, Denmark) until the center mark was reached [28]. Sectioning and polishing were performed by the same operator (V.B.) for standardization. The sectioned specimens were cleaned using a steam cleaner (Wasi-Steam Classic II; Wassermann Dental-Maschinen, Hamburg, Germany) and placed in distilled water at room temperature, which was controlled at 23°C, to prevent desiccation and maintain the dimensional stability of the samples.

After 24 h, the specimens were removed from the distilled water and air-dried. The specimens were stained with 0.1% toluidine blue in 30% alcohol for 24 h to visualize the cement space [34]. To remove excess dye, the specimens were dipped in 50% isopropyl alcohol, rinsed with distilled water, and air-dried for 10 min.

**Figure 7:**
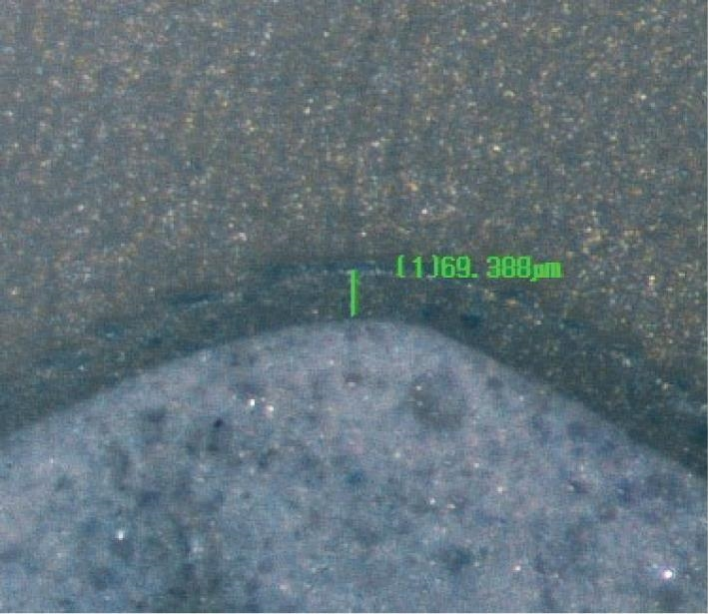
Measurement of internal cement thickness after sectioning of a bonded onlay under a stereomicroscope.

AMD and ICT were measured under an optical stereomicroscope (Nikon SMZ800N; Nikon, Tokyo, Japan) with 6.0x magnification. An example of this measurement is shown in Figure 7, and a schematic view of the AMD and ICT measurement is shown in Figure 1. The measurement points were selected based on the literature [3, 9–11] and are shown in Figure 6. AMD was measured at the buccal, palatal, and mesial sites. For ICT, five measurements were averaged in the vertical section, while four were averaged in the horizontal section. Two independent observers evaluated the measurements under the stereomicroscope. If an error in the measurements was identified, the measurement was repeated for the specimen.

The measurement data were analyzed using analysis software (IBM SPSS Statistics, v27; IBM, Armonk, NY, USA). The data were cleaned to identify outliers, followed by continuous variable descriptive analyses. As the study aimed to understand the variation in AMD and ICT with preparation design, the outliers were not removed. Bivariate analysis was used to understand the association between the preparation designs and both AMD and ICT. The Shapiro–Wilk normality test was conducted to assess the normality of the data. As the results showed evidence of non-normality, the Mann–Whitney *U* test was used for hypothesis testing. The Mann–Whitney *U* test is used for small sample sizes (*n* < 30) to determine if two independent groups are from the same population [35].

## 3. Results

**Figure 8:**
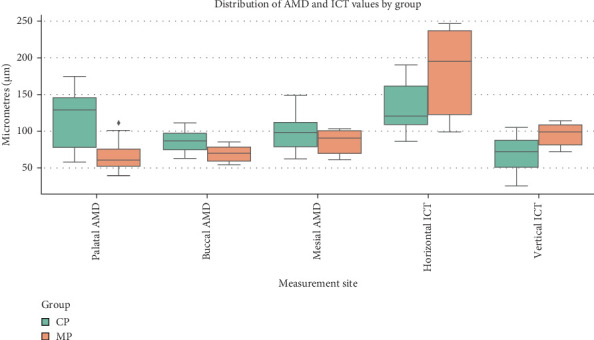
Distribution of AMD and ICT values by group.

Figure 8 shows the results from the descriptive analyses of the continuous data. At the palatal site, MP had an improved AMD (67.78 ± 20.66 µm) in comparison to CP (91.11 ± 50.51 µm). Similarly, the AMD at the buccal site was lower for MP (67.78 ± 10.39 µm) than CP (89.61 ± 36.75 µm), as well as AMD at the mesial site, with a mean of 78.95 ± 12.77 µm for MP, compared to 84.69 ± 40.64 µm for CP. For all AMD sites, MP had a smaller standard deviation than CP.

For ICT in the horizontal section, CP had a reduced mean value (128.18 ± 36.80 µm) compared to MP (170.21 ± 59.31 µm). Similarly, in the vertical section, CP also had a lower ICT (67.87 ± 27.68 µm) than MP (91.46 ± 16.18 µm).

The Mann–Whitney *U* test was used to evaluate whether the two independent groups were from the same population. The results of the Mann–Whitney *U* test are depicted in Table 1. The mean rank showed that CP had increased AMD compared to MP at all sites. This result was statistically significant for palatal AMD (*U* = 25.00; *p*=0.0035) and buccal AMD (*U* = 16.00; *p*=0.006) but not significant for mesial AMD (*U* = 36.50; *p*=0.193).

In contrast, the mean rank showed that CP had reduced ICT compared to MP; however, this result was only statistically significant for the vertical section (*U* = 18.00; *p*=0.009) and not the horizontal section (*U* = 33.00; *p*=0.121).

## 4. Discussion

Based on the findings of this study, the null hypothesis, that no statistically significant difference would be found in an alternative preparation design for bonded onlays in relation to AMD and ICT, was rejected, as the MP presented statistically significant superior AMD at palatal and buccal sites but increased ICT when compared to CP in the vertical section.

To the best of the author's knowledge, no published studies have investigated the effect of preparation design and the marginal and internal fit of 3D-printed restorations. Similar studies using milling protocols and materials have shown that preparation design influences marginal and internal discrepancies [9–12, 20], which is consistent with the results obtained from the present study. However, differences in materials and manufacturing methods should be considered when comparing these results. Studies have shown that milled monolithic zirconia crowns exhibit a statistically significant higher trueness than 3D-printed crowns, likely due to the subtractive manufacturing process, which introduces minimal distortion [21, 36, 37]. In contrast, 3D-printed resins are subject to polymerization shrinkage, and other printing parameters can affect dimensional accuracy [22, 24, 25]. However, both methods show high precision that is compatible with clinical use [37]. As the aim of the present feasibility study was to investigate the MP design on marginal and internal fit, a methacrylate-based provisional resin was used to fabricate the onlays. The principle of using mechanical guidance features to provide a defined path of insertion is applicable across materials; therefore, milled or 3D-printed ceramics may show an amplified result of the improved marginal adaptation seen in this study, due to their higher dimensional stability compared to 3D-printed resins. However, further investigation using these definitive materials is needed to confirm the consistency of these findings.

In the present study, the MP showed a statistically significant smaller AMD at the palatal (67.68 ± 20.66 µm) and buccal (67.68 ± 10.39 µm) sites compared to the CP at the palatal (91.11 ± 50.51 µm) and buccal (89.61 ± 36.75 µm) sites (*p* < 0.05). These findings are consistent with the previous studies using milled materials [10, 11]. Yang et al. [10] evaluated the effect of two preparation designs: a traditional preparation with a heavy chamfer on the functional cusp and a contra-bevel on the nonfunctional cusp, and a shoulder preparation with uniform cusp reduction. They reported that the traditional preparation produced reduced marginal discrepancy compared to the shoulder preparation. Similarly, Neto et al. [11] reported improved marginal discrepancies for an onlay preparation with an occlusal box compared to a preparation without an occlusal box. In the present study, the MP and CP designs differed only by a cross-shaped groove in the occlusal surface; this feature acts as a positioning device that provides guidance in 3D and a defined path of insertion, thereby reducing the risk of improper seating. In contrast, the CP lacks positive seating areas, which can result in difficulty positioning the onlay during cementation, resulting in increased AMD. These findings contradict previous studies [9, 12, 20], which may be attributed to differences in onlay design and restoration materials, cements, and gap measurement methods.

Marginal gaps between 50 and 120 µm [5, 9, 12, 15, 18, 23] and internal gaps below 200 µm [6, 18, 32] are considered clinically acceptable. However, there is currently no established standard for what constitutes a clinically acceptable marginal fit due to inconsistencies in the terminology used to describe misfit, emerging manufacturing materials, and new methods for assessing fit [17]. Furthermore, the lack of studies on 3D-printed restorations makes it challenging to determine whether these traditional AMD and ICT values apply to 3D-printed resins [26]. As such, further clinical studies are needed. Both CP and MP demonstrated mean AMD values ranging from 67.78 to 91.11 µm, all of which are below the suggested 120 µm threshold.

The present study found that MP produced a statistically significant larger ICT in the vertical section (91.46 ± 16.18 µm) compared to CP (67.87 ± 27.68 µm) (*p* < 0.05). Although ICT was also larger in the horizontal section for MP (170.21 ± 59.31 µm) than CP (128.18 ± 36.80 µm), this was not statistically significant (*p*  > 0.05). This finding aligns with the results reported by Seo et al. [9], who observed that larger internal gaps were linked to increased complexity in the preparation. The complex geometry of the cross-shaped groove can create shadows and distort the reflection of light, reducing the scan trueness of intraoral scanners. This may lead to a thicker internal cement layer [15, 16].

In the present study, MP produced clinically acceptable mean ICT values in both vertical and horizontal sections, except for one specimen with an ICT value of 280.54 µm. All measured values were retained in the final analysis, as none met exclusion criteria based on data entry error or technical artifact. Since the aim of the study was to assess whether the new design improved marginal fit, it was important to preserve the full range of values, including those at the upper end. Furthermore, visual inspection identified no extreme or implausible values. This approach aligns with statistical reporting guidelines, which recommend against removing outliers without strong justification, particularly when the objective is to capture the true variability in performance [38]. This did not affect the overall interpretation of the results, as the *p*-value and effect size indicated that this single result had no significant impact on the findings.

Although moderate effect sizes were observed for vertical ICT (*r* = 0.32), palatal AMD (*r* = 0.32), and buccal AMD (*r* = 0.37), these values indicate a measurable difference between preparation designs in terms of marginal and internal fit. Effect size quantifies the magnitude of difference independently of sample size, offering insight into the strength of the association between variables. While the statistical differences suggest design-related trends, further studies are recommended to validate these findings under broader experimental conditions.

The findings of this study suggest that the MP design resulted in an improved AMD in buccal and palatal sites, while it led to an increase in ICT in the vertical section. Marginal gap is critical to long-term success, as increased AMD is associated with microleakage, secondary caries, endodontic complications, and periodontal disease, which are common causes of restoration failure [1, 2]. Additionally, increased ICT may decrease fracture resistance and retention of the restoration [8]. However, a relatively small increase in ICT was observed with the MP design and was still within the clinically acceptable ranges. Therefore, the improvement in AMD may outweigh the observed increase in ICT, especially since the horizontal ICT did not statistically differ between groups.

A literature search was conducted to determine optimal design parameters for the onlays in this study. Ibrahim et al. [32] found superior marginal and internal fit in crowns with a cement spacer of 0 µm compared to 40 and 80 µm. Although this study used digitally manufactured zirconia crowns, a 0 µm cement spacer was used for our study, as limited research is available regarding the optimal cement spacer for 3D-printed onlays.

Printing parameters such as build angle, position of support structures, and layer proximity to the printing platform may affect the trueness of 3D-printed restorations and ICT [22, 24, 25]. Furthermore, the postcuring method influences the degree of conversion, polymerization shrinkage, and stress accumulation within the restoration, which can indirectly affect the ICT [22]. Morón-Conejo et al. [23] found that the method of postprocessing cleaning affected the marginal and internal fit of 3D-printed crowns. The group that was cleaned using ultrasonic immersion resulted in improved marginal and internal fit compared to the group with the automated cleaning machine, which worked by splashing the onlays. Ultrasonic immersion may be more effective at removing residues on the internal surface, resulting in an improved internal fit. The present study produced comparably smaller AMD and ICT in the vertical section. This may be due to cleaning the onlays with ultrasonic immersion for an extended period.

The conventional method was used to replicate the samples, which is reported to produce casts with greater accuracy than 3D printing [30]. Due to the high dimensional stability and precision of polyvinyl siloxane, the replication process using repeated pourings of the same mold is unlikely to cause any significant change in the dies [31].

There are several methods for evaluating marginal and internal fit. This study used 2D sectioning and direct observation under an optical stereomicroscope after cementation, as previous research has shown that marginal gap size tends to increase after cementation. This approach is simple and provides results that closely reflect real clinical conditions [9, 12, 20]. However, the disadvantages of this method include the destruction of the specimens and a limited number of sections and evaluation points, which may not be representative of the overall fit of the restoration [15]. The present study analyzed three sections in three different planes to mitigate this limitation. The silicone replica technique has similar disadvantages but is nondestructive, with a reduced risk of damaging the samples during measurement [10]. Furthermore, the duplicated material may deform upon removal or during sectioning, affecting measurement reliability [10].

Newer techniques, such as µCT and the triple scan protocol, have been developed to overcome the limitations of direct viewing methods. As these techniques allow the visualization of the samples in 3D, multiple measurements can be made per specimen [15]. Furthermore, measurements can be repeated as the specimen is not damaged. A limitation of µCT is low imaging contrast due to scarce X-ray attenuation features [19]. The technique depends on differences in X-ray attenuation to produce contrast in the images. However, due to their low atomic numbers, ceramics and polymer-based materials have low radiopacity. As a result, the low contrast can cause difficulty distinguishing between the cement and the restoration, preventing accurate visualization of small gaps and measurement of ICT and AMD [19]. The accuracy of the triple scan protocol is also dependent on the quality of the digital scan and the alignment of overlapping 3D files [10]. Nevertheless, a systematic review concluded that these methods produce results with high validity and reliability [15]. In future studies investigating the present study MP design with different materials, these 3D techniques can be utilized to obtain measurements at a greater number of sites to examine the overall fit of the restoration.

A limitation of this study was the in vitro study design, as it may not fully reflect clinical situations; clinical parameters such as intraoral access, presence of adjacent teeth, natural dental tissue, humidity, saliva, and patient movement can influence the trueness of intraoral scanner measurements [14]. Therefore, the suggested MP design must be further tested in vivo to confirm these findings. To confirm these findings, the suggested MP design should be tested with definitive materials and different evaluation methods, such as micro-CT. Furthermore, in vivo studies involving randomized and controlled clinical trials are recommended to assess how the MP performs in clinical situations and whether the improved AMD observed in vitro translates into better clinical outcomes.

Additionally, the restoration was bonded onto a resin replica rather than a natural tooth structure. Although this may affect the bonding properties of the material, the in vitro model provided a standardized method for preparation design, onlay fabrication, and measurement, thereby increasing reliability and reproducibility. The method was also standardized by using only one cement, an intraoral scanner, and design software. As the aim of this study was to investigate the effect of preparation design on AMD and ICT, these variables should not have influenced the results.

## 5. Conclusions

Within the limitations of this current study, it was concluded the following:

1. The MP improved the AMD compared to the CP at buccal and palatal sites, but increased ICT in the vertical section.
2. Both preparation designs had a mean AMD and ICT within clinically acceptable limits.

## Figures and Tables

**Table 1:** Mann–Whitney *U* test results for AMD and ICT data.

Measurement site	Sample	*N*	Mean rank	Sum of ranks	Mann–Whitney *U*	*Z*	Asymp. Sig. (two-tailed)	Effect size (*r*)
Palatal AMD	CP	10	14.00	140.00	25.00	−2.11	0.035*⁣*^*∗*^	0.32
	MP	10	8.27	91.00	—	—	—	—
	Total	20	—	—	—	—	—	—

Buccal AMD	CP	10	14.90	149.00	16.00	−2.75	0.006*⁣*^*∗*^	0.37
	MP	10	7.45	82.00	—	—	—	—
	Total	20	—	—	—	—	—	—

Mesial AMD	CP	10	12.55	125.50	36.50	−1.30	0.193	0.08
	MP	10	9.59	105.50	—	—	—	—
	Total	20	—	—	—	—	—	—

Horizontal ICT	CP	10	9.18	101.00	33.00	−1.55	0.121	0.11
	MP	10	13.82	152.00	—	—	—	—
	Total	20	—	—	—	—	—	—

Vertical ICT	CP	10	7.30	73.00	18.00	−2.61	0.009*⁣*^*∗*^	0.32
	MP	10	14.36	158.00	—	—	—	—
	Total	20	—	—	—	—	—	—

*⁣* ^*∗*^ *p* < 0.05.

## Data Availability

The data that support the findings of this study are available from the corresponding author upon reasonable request.

## References

[1] Abduo J., Sambrook R. J. (2018). Longevity of Ceramic Onlays: A Systematic Review. *Journal of Esthetic and Restorative Dentistry*.

[2] Contrepois M., Soenen A., Bartala M., Laviole O. (2013). Marginal Adaptation of Ceramic Crowns: A Systematic Review. *The Journal of Prosthetic Dentistry*.

[3] Holmes J. R., Bayne S. C., Holland G. A., Sulik W. D. (1989). Considerations in Measurement of Marginal Fit. *The Journal of Prosthetic Dentistry*.

[4] Gavranović-Glamoč A., Ajanović M., Korać S. (2018). Evaluation of the Water Sorption of Luting Cements in Different Solutions. *Acta Medica Academica*.

[5] Reich S., Uhlen S., Gozdowski S., Lohbauer U. (2011). Measurement of Cement Thickness Under Lithium Disilicate Crowns Using an Impression Material Technique. *Clinical Oral Investigations*.

[6] Arezoobakhsh A., Shayegh S. S., Jamali Ghomi A., Hakimaneh S. M. R. (2020). Comparison of Marginal and Internal Fit of 3-Unit Zirconia Frameworks Fabricated With CAD-CAM Technology Using Direct and Indirect Digital Scans. *The Journal of Prosthetic Dentistry*.

[7] May L. G., Kelly J. R., Bottino M. A., Hill T. (2012). Effects of Cement Thickness and Bonding on the Failure Loads of CAD/CAM Ceramic Crowns: Multi-Physics FEA Modeling and Monotonic Testing. *Dental Materials*.

[8] May L. G., Robert Kelly J., Bottino M. A., Hill T. (2015). Influence of the Resin Cement Thickness on the Fatigue Failure Loads of CAD/CAM Feldspathic Crowns. *Dental Materials*.

[9] Seo D., Yi Y., Roh B. (2009). The Effect of Preparation Designs on the Marginal and Internal Gaps in Cerec3 Partial Ceramic Crowns. *Journal of Dentistry*.

[10] Yang Y., Yang Z., Zhou J., Chen L., Tan J. (2020). Effect of Tooth Preparation Design on Marginal Adaptation of Composite Resin CAD-CAM Onlays. *The Journal of Prosthetic Dentistry*.

[11] Neto C. F., Santos G. C., Santos M. J. M. C. (2020). Influence of the Fabrication Technique on the Marginal and Internal Adaptation of Ceramic Onlays. *Operative Dentistry*.

[12] Falahchai M., Babaee Hemmati Y., Neshandar Asli H., Neshandar Asli M. (2020). Marginal Adaptation of Zirconia-Reinforced Lithium Silicate Overlays With Different Preparation Designs. *Journal of Esthetic and Restorative Dentistry*.

[13] Lima F. F., Neto C. F., Rubo J. H., Santos G. C., Moraes Coelho Santos M. J. (2018). Marginal Adaptation of CAD-CAM Onlays: Influence of Preparation Design and Impression Technique. *The Journal of Prosthetic Dentistry*.

[14] Merrill T. C., Mackey T., Luc R., Lung D., Naseem A., Abduo J. (2021). Effect of Chairside CAD/CAM Restoration Type on Marginal Fit Accuracy: A Comparison of Crown, Inlay and Onlay Restorations. *The European Journal of Prosthodontics and Restorative Dentistry*.

[15] Goujat A., Abouelleil H., Colon P., Jeannin C., Pradelle N., Seux D. (2019). Marginal and Internal Fit of CAD-CAM Inlay/Onlay Restorations: A Systematic Review of in Vitro Studies. *The Journal of Prosthetic Dentistry*.

[16] Ammoun R., Suprono M. S., Goodacre C. J., Oyoyo U., Carrico C. K., Kattadiyil M. T. (2020). Influence of Tooth Preparation Design and Scan Angulations on the Accuracy of Two Intraoral Digital Scanners: An in Vitro Study Based on 3-Dimensional Comparisons. *Journal of Prosthodontics*.

[17] Boitelle P., Tapie L., Mawussi B., Fromentin O. (2018). Evaluation of the Marginal Fit of CAD-CAM Zirconia Copings: Comparison of 2D and 3D Measurement Methods. *The Journal of Prosthetic Dentistry*.

[18] Uzgur R., Ercan E., Uzgur Z., Çolak H., Yalçın M., Özcan M. (2018). Cement Thickness of Inlay Restorations Made of Lithium Disilicate, Polymer-Infiltrated Ceramic and Nano-Ceramic CAD/CAM Materials Evaluated Using 3D X-Ray Micro-Computed Tomography. *Journal of Prosthodontics*.

[19] Rashidi A., Olfatbakhsh T., Crawford B., Milani A. S. (2020). A Review of Current Challenges and Case Study Toward Optimizing Micro-Computed X-Ray Tomography of Carbon Fabric Composites. *Materials*.

[20] Athab Hasan S., Mohammed-Hussain Abdul-Ameer Z. (2023). Effect of Three Different Preparation Designs on the Marginal Adaptation of Indirect Overlay Restoration Fabricated From Lithium Disilicate Ceramic Material: An in-Vitro Comparative Study. *The Saudi Dental Journal*.

[21] Lerner H., Nagy K., Pranno N., Zarone F., Admakin O., Mangano F. (2021). Trueness and Precision of 3D-Printed Versus Milled Monolithic Zirconia Crowns: An in Vitro Study. *Journal of Dentistry*.

[22] Sampaio C. S., Niemann K. D., Schweitzer D. D., Hirata R., Atria P. J. (2021). Microcomputed Tomography Evaluation of Cement Film Thickness of Veneers and Crowns Made With Conventional and 3D Printed Provisional Materials. *Journal of Esthetic and Restorative Dentistry*.

[23] Morón-Conejo B., Berrendero S., Bai S., Martínez-Rus F., Pradies G. (2024). Fit Comparison of Interim Crowns Manufactured With Open and Proprietary 3D Printing Modes Versus Milling Technology: An in Vitro Study. *Journal of Esthetic and Restorative Dentistry*.

[24] Alghauli M. A., Aljohani R., Almuzaini S., Aljohani W., Almutairi S., Alqutaibi A. Y. (2025). Accuracy, Marginal, and Internal Fit of Additively Manufactured Provisional Restorations and Prostheses Printed at Different Orientations. *Journal of Esthetic and Restorative Dentistry*.

[25] Alharbi N., Osman R., Wismeijer D. (2016). Factors Influencing the Dimensional Accuracy of 3D-Printed Full-Coverage Dental Restorations Using Stereolithography Technology. *The International Journal of Prosthodontics*.

[26] Shin H., Kang Y.-J., Kim H., Kim J.-H. (2025). Effect of Cement Space Settings on the Marginal and Internal Fit of 3D Printed Definitive Resin Crowns. *The Journal of Prosthetic Dentistry*.

[27] Yu H., Zhao Y., Li J., Luo T., Gao J., Liu H. (2019). Minimal Invasive Microscopic Tooth Preparation in Esthetic Restoration: A Specialist Consensus. *International Journal of Oral Science*.

[28] Bennani V., Aarts J. M., Senthilkumar A. (2024). Effect of a Modified Laminate Veneer Preparation Design on Absolute Margin Discrepancy and Marginal Overhang. *The Journal of Prosthetic Dentistry*.

[29] Chu J., Bennani V., Aarts J. M., Chandler N., Lowe B. (2018). The Effect of Different Geometric Shapes and Angles on the Fracture Strength of IPS e.max Computer-Aided Designed Ceramic Onlays: An in Vitro Study. *Journal of Conservative Dentistry*.

[30] Park M.-E., Shin S.-Y. (2018). Three-Dimensional Comparative Study on the Accuracy and Reproducibility of Dental Casts Fabricated by 3D Printers. *The Journal of Prosthetic Dentistry*.

[31] Niekawa C. T., Kreve S., A’vila G. B., Godoy G. G., Eduardo Vieira da Silva J. R., Dias S. C. (2017). Analysis of the Mechanical Behavior and Surface Rugosity of Different Dental Die Materials. *Journal of International Society of Preventive and Community Dentistry*.

[32] Ibrahim H., El Kateb M., Morsy N. (2024). Effect of Modifying Occlusal Cement Spacer on the Fit Accuracy of Digitally Manufactured Zirconia Crowns. *The Journal of Prosthetic Dentistry*.

[33] Lee B.-I., You S.-G., You S.-M., Kang S.-Y., Kim J.-H. (2021). Effect of Rinsing Time on the Accuracy of Interim Crowns Fabricated by Digital Light Processing: An *In Vitro* Study. *The Journal of Advanced Prosthodontics*.

[34] Petrovic B., Pantelinac J., Capo I., Miljkovic D., Popovic M., Penezic K. (2021). Using Histological Staining Techniques to Improve Visualization and Interpretability of Tooth Cementum Annulation Analysis. *International Journal of Morphology*.

[35] MacFarland T. W., Yates J. M., MacFarland T. W., Yates J. M. (2016). Mann–Whitney U Test. *Introduction to Nonparametric Statistics for the Biological Sciences Using R*.

[36] Wang W., Yu H., Liu Y., Jiang X., Gao B. (2019). Trueness Analysis of Zirconia Crowns Fabricated With 3-Dimensional Printing. *The Journal of Prosthetic Dentistry*.

[37] Mosaddad S. A., Peláez J., Panadero R. A., Ghodsi S., Akhlaghian M., Suárez M. J. (2025). Do 3D Printed and Milled Tooth-Supported Complete Monolithic Zirconia Crowns Differ in Accuracy and Fit? A Systematic Review and Meta-Analysis of in Vitro Studies. *The Journal of Prosthetic Dentistry*.

[38] Osborne J. W., Overbay A. (2004). The Power of Outliers (and Why Researchers Should Always Check for Them). *Practical Assessment, Research, and Evaluation*.

